# The Impact of Climate Change on the Potential Distribution of Agricultural Pests: The Case of the Coffee White Stem Borer (*Monochamus leuconotus P.*) in Zimbabwe

**DOI:** 10.1371/journal.pone.0073432

**Published:** 2013-08-27

**Authors:** Dumisani Kutywayo, Abel Chemura, Winmore Kusena, Pardon Chidoko, Caleb Mahoya

**Affiliations:** 1 DR&SS, Coffee Research Institute, Chipinge, Zimbabwe; 2 Dept of Environmental Science and Technology, Chinhoyi University of Technology, Chinhoyi, Zimbabwe; 3 GES Department, Midlands State University, Gweru, Zimbabwe; Natural Resources Canada, Canada

## Abstract

The production of agricultural commodities faces increased risk of pests, diseases and other stresses due to climate change and variability. This study assesses the potential distribution of agricultural pests under projected climatic scenarios using evidence from the African coffee white stem borer (CWB), *Monochamus leuconotus* (Pascoe) (Coleoptera: Cerambycidae), an important pest of coffee in Zimbabwe. A species distribution modeling approach utilising Boosted Regression Trees (BRT) and Generalized Linear Models (GLM) was applied on current and projected climate data obtained from the WorldClim database and occurrence data (presence and absence) collected through on-farm biological surveys in Chipinge, Chimanimani, Mutare and Mutasa districts in Zimbabwe. Results from both the BRT and GLM indicate that precipitation-related variables are more important in determining species range for the CWB than temperature related variables. The CWB has extensive potential habitats in all coffee areas with Mutasa district having the largest model average area suitable for CWB under current and projected climatic conditions. Habitat ranges for CWB will increase under future climate scenarios for Chipinge, Chimanimani and Mutare districts while it will decrease in Mutasa district. The highest percentage change in area suitable for the CWB was for Chimanimani district with a model average of 49.1% (3 906 ha) increase in CWB range by 2080. The BRT and GLM predictions gave similar predicted ranges for Chipinge, Chimanimani and Mutasa districts compared to the high variation in current and projected habitat area for CWB in Mutare district. The study concludes that suitable area for CWB will increase significantly in Zimbabwe due to climate change and there is need to develop adaptation mechanisms.

## Introduction

The agricultural sector, due to its size and sensitivity, is at risk from global climate change with a projected average global yield loss of up to 50% by 2050 [1], [2]. Tropical countries, which already have low productivity due to other development challenges are vulnerable to the risks of climate change [3]. In Southern Africa, agriculture is very important for economic development, poverty alleviation, employment and food security, yet vulnerability to climate risks is considered very high because of heavy reliance on natural resources particularly water and rainfed traditional agricultural systems [4], [5]. Few studies have been conducted to analyse the effect of biotic stresses on plantation crops such as coffee, citrus, oil palm, rubber and others despite the fact that these are important for domestic consumption and economic growth [6], [7]. Coffee (*Coffea arabica L.*) production is an important economic activity in over 70 countries across 4 continents with an estimated annual trade value of over twenty billion US dollars and employing millions in the value chain [8]–[10].

Climate change is projected to have many effects on agricultural productivity in the tropical areas [11]–[13]. The coffee value chain will be affected from producers and farm workers to those involved with coffee distribution and marketing [14], [15]. Rainfall becoming unreliable and temperature increases are expected to reduce the suitability of many coffee producing zones as currently suitable areas become marginal [14], [16], [17]. The incidence and severity of some coffee pests and diseases such as the coffee berry borer *(Hypothenemus hampei* (Ferrari)) and coffee leaf rust (*Hemileia vastatrix* (B & Br)) are projected to increase, reducing yield and quality and increasing production costs [8], [18], [19]. These effects will be profound for the smallholder coffee farmers in Africa, Asia and Latin America who supply up to 70% of global coffee, rely in many cases solely on coffee as a unique and legal source of livelihoods, and lack both coping and alternative economic options [10], [20].

In Zimbabwe, only Coffee arabica is produced mainly in the eastern highlands districts of Chipinge, Chimanimani, Mutare and Mutasa, and in the northern parts of the country in Guruve, Harare and Mhangura [21], [22]. Coffee is regarded as the second most profitable crop in Zimbabwe after flu-cured tobacco [13], [23]. Pests of economic significance for both smallholder and large scale coffee producers are the African coffee white stem borer (*Monochamus leuconotus* (Pascoe)), the coffee leaf minor (*Leucoptera meyricki* (Ghesquiére)) and the antestia bug (*Antestiopsis orbitalis* (Kirk)); while major diseases include coffee leaf rust, Fusarium bark disease (*Fusarium lateritium*) and the coffee berry disease (*Colletorichum kahawae* (J.M.Waller & Bridge)) [21], [24], [25].

The coffee white stem borer belongs to the family Cerambycidae and sub-family Lamiinae [26]. The adult is a beetle up to 30 mm long with black patches on a brown back and has long horns that are twice the body length in males [24], [27]. Although the adult's feeding activities may damage buds, shoots and stem bark, it is the feeding activities of the larvae on the core and lower parts of the plant that are associated with reduced productivity and economic losses. The adults lay eggs on the bark of the coffee plant and when hatched the larvae bore into the bark causing ring barking and choking of the plant system with frass, which disturbs uptake of metabolites [27]. The presence of CWB is detected by yellowing of leaves, signs of ring barking, exit holes and frass. CWB attack leads to stunted growth, wilting, dieback and reduced yields on affected plants [24], [28]. The complete life cycle of the CWB can be up to two years with the larval stage taking about 20 months [24], [27].

The CWB is the most serious pest of coffee in Zimbabwe with yield losses of up to 25% and its prevalence and severity has been increasing in recent decades [28]. The CWB was noted to be more prevalent on smallholder farms with incidence of up to 70% and all coffee varieties grown in Zimbabwe were susceptible to the pest. Factors such as age of the coffee plant, mulching and shade are important in influencing both the incidence and severity of CWB on coffee farms in Zimbabwe. The yield losses due to CWB were highest in Chipinge district with 25%, which was above the average national yield loss of 15% [28]. The common management practices for the CWB in Zimbabwe are picking and killing adults as well as uprooting and burning infested plants [28]–[30]. Chemical control measures for control of CWB include chlorpyrifos, endosulphan, fipronil, methomyl and monochrotophos [28], [31]. These are applied as stem banding treatments by a knapsack sprayer or paint brush up to 90 cm above the ground targeting the female adults, early instar larvae and eggs [21], [28], [31]. However many of these chemicals are already either banned or listed and will be phased out.

Adult emergence of the CWB begins in late November to December, peaks in January and ends in April, closely matching the rainfall and temperature patterns of Zimbabwe [27], [29], [30]. This indicates that there is a weather dependence on the life cycle and emergence patterns of CWB. Since there are significant relationships between the life cycles of insects and weather variables, it is possible to predict population dynamics and incidences of pests for production areas over long periods of times [8], [32]. For example, cold temperatures disrupt egg development, survival of larvae and result in mortality of emerged adults [33], [34]. On the other hand, warm temperatures are favourable for the successful completion of the pest life cycle while increasing host plant susceptibility to pest attack [8], [18], [32], [33].

Species distribution modeling (SDM) is important in determining realized niches of species and is useful for planning and understanding the impacts of climate on the habitat suitability [35], [36]. SDMs are also referred to as habitat models, niche-models and/or climate envelope models. The major assumption of these models is that the potential occurrence areas of a species can be spatially predicted using characteristics of known occurrence or non-occurrence within its range [36], [37]. Over the years many SDMs have been developed and they vary in terms of complexity and in the environmental variables used to predict species distributions. Among the most commonly used SDMs are Boosted Regression Trees (BRT), Generalized Linear Models (GLM), Generalized Additive Models, Random Forests (RF), and the Maximum Entropy (Maxent) [37]–[39]. The applicability and utility of SDMs has been widely reviewed and explored [35], [37], [39]–[42].

With the risks of climate change becoming more apparent in some sectors, there is a need to develop and test tools that can be useful in understanding the impact of global warming, climatic variability and related phenomena on the potential of agricultural systems to maintain or improve productivity and profitability, sustain livelihoods and safeguard the integrity of landscapes [2], [6]. This is especially important in understanding where, when and how efficient and systematic response mechanisms can be developed in advance. The purpose of this study was to use species distribution models to understand the distribution of CWB and factors that influence its distribution in Zimbabwe. In addition, the study aims to apply the models to predict the changes in the distribution of the pest under projected future climatic conditions. We apply an SDM approach to understand the distribution of CWB from known presence and absence points under current and projected climatic conditions. The results are important for planning efficient and systematic control of the pest in coffee and as such two models are used in order to ascertain reliability of the predictions. We consider this study important as a baseline in giving indications of the impact of climate change on distribution of invertebrate species of agricultural importance. Despite indications from other studies, this study was carried out on the assumption that with successful adaptation technologies such as irrigation, moisture conservation and climate-ready varieties, the current coffee zones in Zimbabwe will not change and therefore focuses only on the CWB risk in these areas.

## Materials and Methods

### Study area

**Figure 1:**
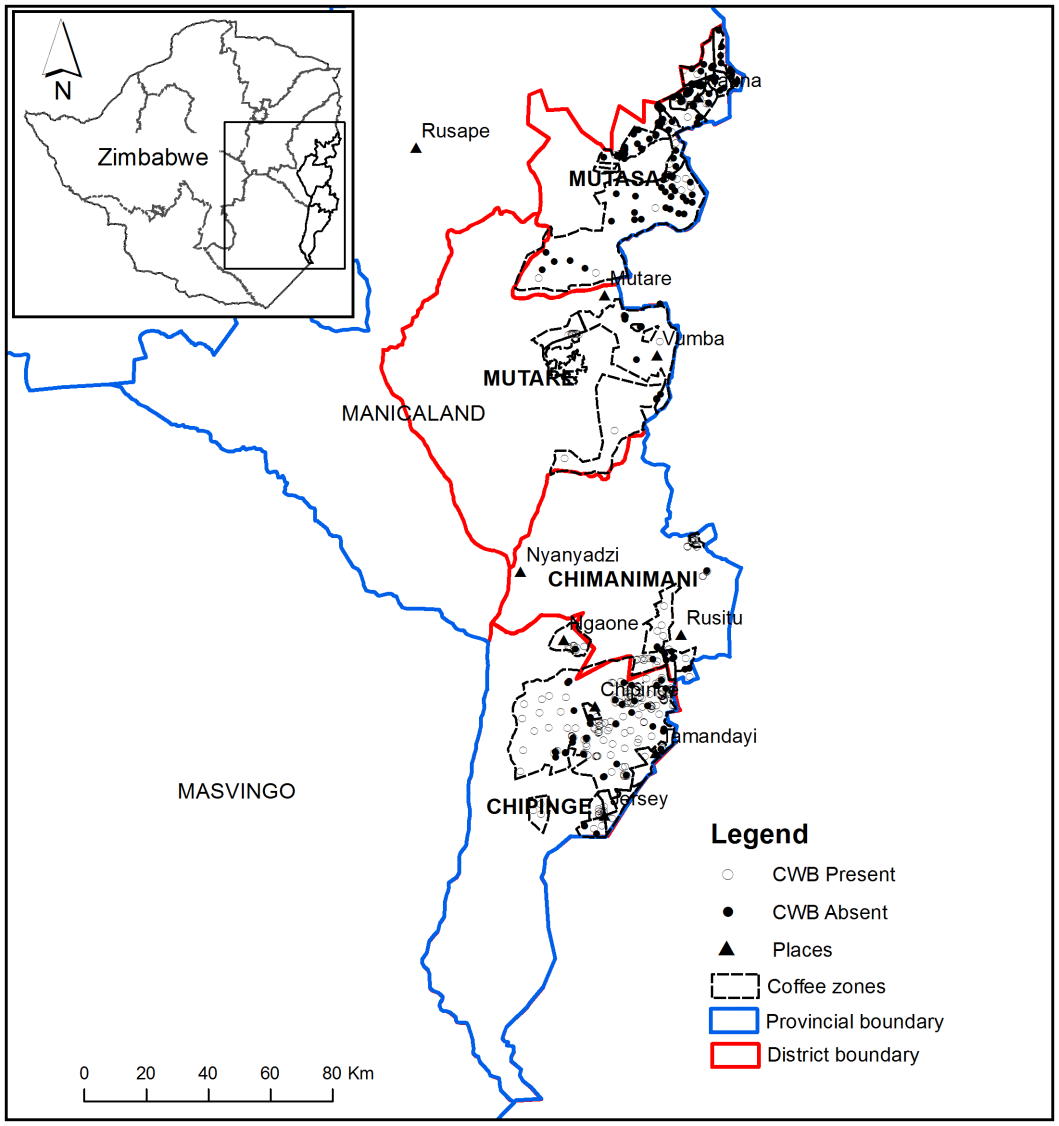
Map of the study areas showing the districts and coffee zones in Zimbabwe. The closed and open circles indicate locations where CWB was sampled as present and absent respectively. A context map is given to provide location of the study area in Zimbabwe.

The study was carried out in four agricultural districts in the Manicaland province of Zimbabwe (Chipinge, Chimanimani, Mutare and Mutasa), also known as the eastern highlands of Zimbabwe (Figure 1). These districts also cover mainly agro-ecological region 1, which is highly valued for its contribution to agricultural productivity in Zimbabwe. These districts represent the main coffee production zone in Zimbabwe in terms of numbers of farmers and area under production. The eastern highlands area is characterized by high rainfall (over 1000 mm per anum). The highest elevation for a coffee farm in this study was at an altitude of 1661 masl in the Chipinge district while the lowest was at 631 masl in Mutasa District. The main coffee production system is sun-coffee under intensive management and different degrees of shading and cover cropping especially in the smallholder sector.

### Ethics

While no specific permits were required to carry out the field surveys, permission was sought from each resident coffee farmer before field sampling. Farms where the resident farmer or his/her representative was not available at the time of the survey were skipped. No field studies involved endangered or protected species.

### Occurrence data

Data on the occurrence (presence/absence) of the CWB were collected from all coffee producing districts in 2003 through biological field surveys of coffee farms. At each farm the xy coordinates (in WGS84 datum) were collected using a global position system (Garmin eTrex Vista®) together with the presence or absence of CWB on 30 coffee trees selected at random. This dataset was converted to presence and absence data for each of the sites. The presence of CWB was determined by checking signs of CWB damage (larval damage frass, adult emergence holes and adult feeding wounds) and/or the presence of adults in the coffee trees between December and March. A total of 929 points were collected during field surveys but 259 points were rejected for the purpose of this study because they fell within the same environmental data pixel (1 km×1 km). A total of 670 unique points were used in this study with 70% (N = 469) of the points randomly subset for use in model calibration and the remaining 30% (N = 201) for accuracy assessment. The training data gave a model that was then used to predict the occurrence of the CWB across sites that had not been sampled. Only actual field collected data were used in evaluating the performance of the modeling.

The occurrence data represented the occurrence of CWB in coffee farms but were assumed to represent suitability of CWB for both farms and surrounding natural habitats. All data were used because the effect of current chemical and physical control measures was considered insignificant since a number of indicators were used to determine presence or absence of CWB. Chemical control is done in November (before the rains) [24], [30] and thus some of the adult CWB that determined presence on coffee farms could be from wild hosts and independent of any on-farm management practices. In many of the sites (especially in smallholder coffee farms), the application of chemical control methods is not always effective because of use of incorrect rates and mistiming of applications.

### Environmental layers

For the current and projected climate conditions, six BIOCLIM layers were obtained from the WorldClim database (http://www.worldclim.org) [43]. These six layers were chosen based on their potential relationship to coffee production as well as to the CWB emergence patterns (Table 1) [30]. The data were available in 1 km^2^ grids and are interpolated from weather station recordings. For the predicted scenario for 2080, this study used the predictions from the Hadley Centre Coupled Model version 3 (HadCM3) A2A scenario developed by the Hadley Centre for Climate Prediction and Research WorldClim database (http://www.worldclim.org/futdown.htm). This scenario assumes an above average increase in atmospheric CO_2_ but not as much as the worst case scenario [33].

**Table 1:** Environmental layers used for current and predicted climate in the study.

Code	Factor	Units
Bio1	Annual mean temperature	°C
Bio4	Temperature seasonality	°C (×100)
Bio5	Maximum temperature of warmest month	°C
Bio8	Mean temperature of the wettest month	°C
Bio15	Precipitation seasonality	mm
Bio16	Precipitation of wettest quarter	mm

The HadCM3 model was chosen for this analysis because it was reported to have reliable ranges in climate predictions for Africa when tested against observed data [7], [8]. The environmental layers were clipped to the coffee producing districts of Zimbabwe in ArcGIS Desktop Release 10 [44]. The Variance Inflation Factor (VIF) was used to determine whether there were correlations between the predictor variables whose correlations were above 0.5 [45]. Only one variable had a correlation coefficient less than 0.5 (precipitation seasonality (Bio15)) with other variables. However, VIF showed that there was no serious multicollinearity between the variables except annual mean temperature (Bio1) and mean temperature of the wettest month (Bio8) whose VIF were above 10 but were deemed ecologically important for the CWB distribution [46].

### Modeling approach

In order to obtain the current and projected distribution of a species, many modeling techniques can be used depending mainly on the availability of data and applicability of the output to the biology of the species being modelled [35]. In this study, BRT and GLM model calculations were performed in R environment 2.13.0 with the ‘dismo’, ‘raster’, ‘maptools’ ‘PresenceAbsence’, ‘gbm’ and associated packages [47] to obtain the current and projected distribution of CWB within the four districts using the selected environmental variables [48].

The BRT model (also referred to as stochastic gradient boosting) is considered among the most reliable spatial distribution models compared to other approaches especially where presence and absence data are available [39], [49], [50]. The BRT fits complex non-linear functions to data by combining regression trees (linking the response variable to predictor variables through recursive binary splits) and boosting (additive combinations of models for improved performance) [38], [49]. The strength of BRT is in capturing interactions of prediction layers and dealing with sharp discontinuities [40], [49]. The BRT has an improved predictive performance as it reduces over-learning or over-fitting common in other approaches as it can assume a linear, curvilinear or non-linear function where the choice of error distribution includes normal, binomial or Poisson distribution [49], [50]. It is also robust to the effects of multicollinearity as it can handle data with outliers, a large number of predictors and missing predictor values [49], [51]. A learning rate (contribution of each tree to the growing model) of 0.01 and a bag fraction (the percentage of data that the model uses for each step) of 0.5 was used for the BRT function.

The GLM was used comparatively with the BRT because it is among the most common approaches to species distribution modeling [35], [37]. GLM uses parametric functions to predict the dependent variable from linear, quadratic or cubic combinations of explanatory variables [38], [52]. In this study, stepwise model selection using the Akaike Information Criterion (AIC) was used [48], [53]. The GLM is widely used in ecological modeling because it is able to deal with data with different error structures associated with presence-absence data [35], [52]. Diagnostic plots were used to assess conformity of residuals to parametric assumptions.

Spatial autocorrelation, a major issue in regression based modeling, was assessed using the Moran's *I* correlogram to determine whether there was a spatial pattern in the residuals of models with distance [38]. The relative influence (RI) was calculated for each of the predictor variables (β_x_) in the model through deviance decomposition per degree of freedom ([51], Equation 1)
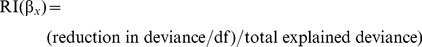
(1)


The outcome of the modeling was the probability of CWB occurrence for each pixel ranging between 0 (less likely to occur) and 1(most likely to occur). This outcome provided the spatial distribution of the current and future distribution of CWB in the study area. In addition to the probability maps, it was considered important to have a categorical map that can be used to evaluate model prediction and delineate areas as being suitable or not suitable habitats of CWB [37], [54]. This is achieved by setting a threshold probability value above which the species will be predicted to occur and below which it is predicted not to occur. Although there are discussions about how this threshold should be selected [37], [53]–[56], we found it most appropriate to use a fixed value of 0.5 (50%) as our threshold. This was because optimising thresholds produced different optimal thresholds for the BRT and the GLM which made model comparison appear unfair. In addition, none of the methods of selecting a threshold tested provided a consistent statistically better outcome than the 0.5 threshold in terms of sensitivity, specificity, Kappa and percent correctly classified (PCC). The 50% threshold is also commonly used as a threshold in species distribution modeling [57].

## Results

### Relative importance of environmental predictors

The CWB distribution was shown to have a greater response to precipitation factors than to temperature-related factors in both the BRT and the GLM. For the BRT model, average precipitation of the wettest quarter was the best predictor of the distribution of CWB (45.6%) followed by precipitation seasonality with a relative importance of 24.2%. In the BRT model temperature seasonality was also a significant predictor of CWB distribution with an RI of 18.1% (Table 2). In the GLM model however, precipitation seasonality was the best predictor of CWB distribution. Precipitation seasonality accounted for 75.7% of the CWB distribution for the GLM model, with the variables precipitation seasonality and precipitation of the wettest quarter combined accounting for 88.2%. For both the BRT and the GLM, mean temperature of the wettest month was the least important parameter (Table 2).

**Table 2:** Relative importance of factors in each model.

Variable	BRT	GLM
Bio16	45.6	12.5
Bio15	24.2	75.7
Bio4	18.1	0.3
Bio5	6.0	4.9
Bio1	3.2	6.3
Bio8	2.9	0.2

The optimum numbers of trees for the BRT model were 300 and they had a training data correlation of 0.652, a ROC of 0.876 and a discrimination mean of 0.815. The final GLM model from the stepwise regression had three parameters; precipitation seasonality, precipitation of the wettest quarter and annual mean temperature. Precipitation seasonality was highly significant (*P*<0.001) while the other variables were significant (*P*<0.05) in predicting CWB distribution.

### Current distribution of CWB

**Figure 2:**
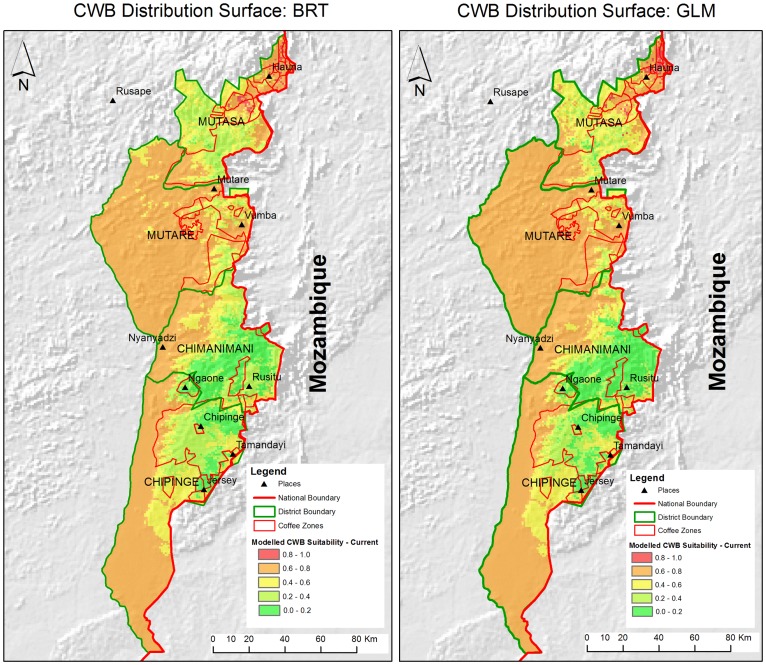
Probability of occurrence of CWB that determines its distribution surface in the four Districts. Figure 2(a) shows the probability of CWB occurrence for each area obtained from BRT model. Figure 2(b) shows the probability of CWB occurrence for each area obtained from GLM.

The BRT and GLM models showed the potential distribution of the CWB under current climatic conditions in Zimbabwe. Mutasa and Chipinge districts have the largest coffee producing areas suitable for CWB while Chimanimani has the least. The models showed that northern parts of the eastern highlands (Mutasa and Mutare districts) have areas that are more suitable for the CWB than the southern districts of Chimanimani and Chipinge (Figure 2a and 2b).

**Figure 3:**
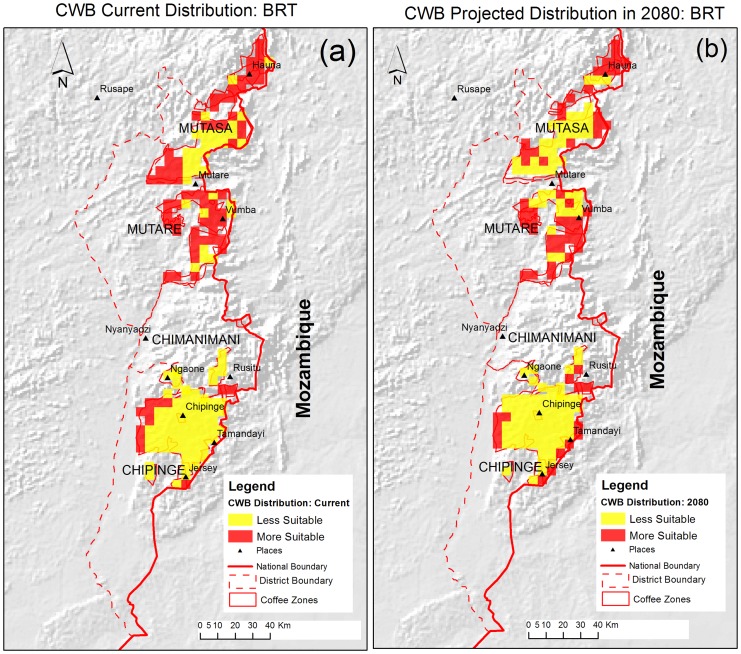
Distribution of CWB in coffee zones from BRT Model. Figure 3(a) shows the current distribution of CWB from the BRT model. Figure 3(b) shows the projected distribution of CWB from the BRT. The red zones are areas with a probability of occurrence above 0.5 indicating CWB is likely to occur and the yellow zones are areas that have a probability below 0.5 indicating CWB is less likely to occur.

**Figure 4:**
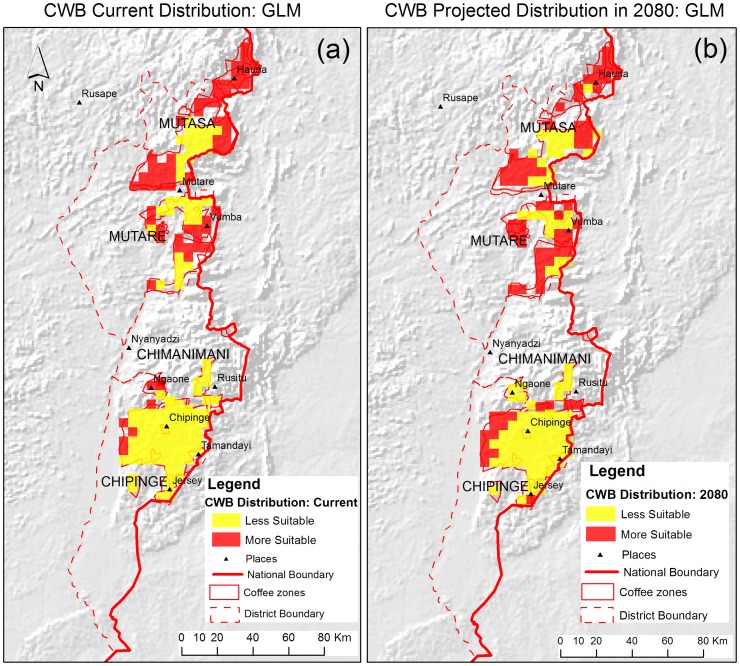
Distribution of CWB in coffee zones from GLM. Figure 4(a) shows the current distribution of CWB from the GLM. Figure 4(b) shows the projected distribution of CWB from the GLM. The red zones are areas with a probability of occurrence above 0.5 indicating CWB is likely to occur and the yellow zones are areas that have a probability below 0.5 indicating CWB is less likely to occur.

The current suitable areas for CWB according to the BRT model are shown in Figure 3a while the CWB suitable areas according to the GLM are in Figure 4a. Mutasa district had the largest highly suitable area for CWB under current climatic conditions according to both the BRT and the GLM with 88 999 ha and 107 877 ha respectively (Table 3). High risk areas are concentrated on the northern parts of the districts (Figure 3a and 4a). For Mutasa district, 58.9% and 71.4% of the area under coffee were found to be highly suitable for CWB by the BRT and the GLM models respectively. This was the highest area suitable for CWB in all the four districts (Table 4). The BRT and the GLM concurred on the area and spatial distribution of CWB risk for Chipinge district with BRT predicting 26 969 ha (14.3%) while the GLM showed 26 878 ha (14%). The western parts of Chipinge district had better conditions suitable for CWB compared to the eastern parts in both models. However, the BRT showed that the area around Jersey had a high risk of CWB (Figure 3a) while the GLM considered that area to have low risk (Figure 4a).

**Table 3:** Current and projected area suitable for CWB obtained from the BRT and the GLM model (ha).

District	BRT		GLM	
	Current	2080	Current	2080
Chipinge	26969	30252	26878	35060
Chimanimani	5394	7806	2697	8098
Mutare	24272	62347	45848	49423
Mutasa	88999	66142	107877	83605

**Table 4:** Area and percentage change in area suitable for CWB with climate change. The mean is the average of the GLM and BRT models.

	Current area(ha)			Projected area(ha): 2080			% Change		
District	GLM	BRT	Mean	GLM	BRT	Mean	GLM	BRT	Mean
Chipinge	26878	26969	26924	35060	30252	32656	30.4	12.2	17.6
Chimanimani	2697	5394	4045	8098	7806	7952	200.3	44.7	49.1
Mutare	45848	24272	35060	49423	62347	55885	7.8	156.9	37.3
Mutasa	107877	88999	98438	83605	66142	74873	−22.5	−25.7	−31.5
National	183300	145634	164467	176186	166547	171366	−3.9	14.4	4.0

The models disagreed on the distribution of CWB in Mutare district with the GLM showing 47.1% more area suitable for CWB than the BRT under current climate conditions (Figure 3a and 4a). Chimanimani district had a relatively small area currently under coffee and the risk of CWB was low for the 80% and 90% of that area according to BRT and the GLM respectively (Figure 3a and 4a). Notably, the area that was considered to have a high risk according to the BRT was different from the area that was considered to have a high risk by the GLM in Chimanimani district while for other districts the areas generally corresponded. In total, 31.0% (145 634 ha) of the area in Zimbabwe was considered to have a high risk of CWB under current conditions by the BRT model compared to 39.5% (183 300 ha) according to the GLM.

### Model accuracy assessment

**Figure 5:**
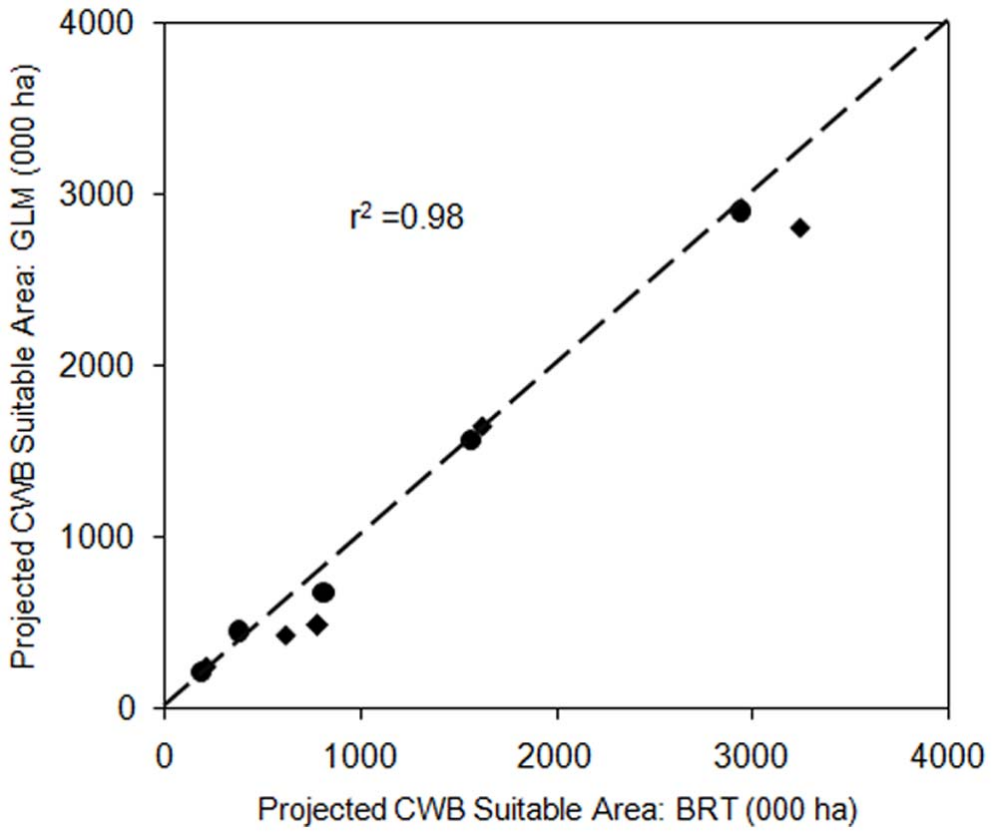
Comparison of area suitable for CWB for each district as determined by BRT and GLM under current (•) and projected (♦) climatic conditions.

Evaluation of the model performance using occurrence data under the current climate scenario showed that both models had a good performance in predicting the distribution of CWB (AUC>0.75). The performance statistics of the two models were close for specificity and Area under the Curve (AUC) while they differed for model sensitivity and kappa with the GLM being inferior to the BRT in both cases (Table 5). There was high agreement between the GLM and BRT predictions of areas suitable for CWB under current and predicted climatic conditions (r^2^ = 0.98, Figure 5). The BRT predictions were below GLM predictions in area suitable for CWB under projected climate.

**Table 5:** Evaluation of model performance.

Statistic	BRT	GLM
Sensitivity	0.81	0.76
Specificity	0.72	0.71
AUC	0.79	0.78
Kappa	0.53	0.47

### Projected distribution of CWB by 2080

The distribution of CWB under projected climatic conditions in the Chipinge district showed that 16.2% of the district (30 252 ha) will have a high risk of CWB infestation according to the BRT (Figure 3b). The GLM predicted 18.3% (35 060 ha) to become suitable for CWB in 2080 for Chipinge district, an increase of 2.1% compared with the BRT prediction (Figure 3b). A total area of 7 806 ha (29.3%) and 8 098 ha (27.3%) were projected to be highly suitable for CWB infestation by the BRT and the GLM respectively in Chimanimani district (Figure 3b and 4b). For the Mutare district, projections by the BRT were that 62.3% of the area will be at risk of CWB under future climatic conditions while the GLM projected 55.4% of the area.

The Mutare district is the only district where the BRT projected that more than half the area would be at high risk for infestation. Forty-five per cent of Mutasa district (66 142 ha) will be highly suitable for CWB infestation under projected climatic conditions for 2080 according to the BRT compared to 83 605 ha (55.4%) according to the GLM. Using the BRT, a total of 166 547 ha (36.2%) are projected to have a high risk and this agrees with 37.8% predicted by the GLM. For the Chipinge, Chimanimani and Mutasa districts, the GLM predicted more area to be at risk of CWB while for Mutare district the BRT projected more area than the GLM (Figure 3b and 4b).

The BRT model predicted high risk of infestation in both the east and west of Chipinge (Figure 3b), while the GLM predicted high risk exclusively in the west (Figure 4b). According to the BRT model, the western parts and some areas in the eastern parts of Chipinge district will have a higher risk of CWB (Figure 4b) In the Chimanimani district, the BRT and the GLM predict that southern parts of the district will have high risk of CWB, although the BRT extended the area to central parts of the coffee areas in the district. The eastern, western and pockets of the northern parts of the Mutare district will be at high risk of CWB according to both models. For Mutasa district, the models agree on the distribution of CWB in the northern parts and eastern-central parts of the district while they differ on the distribution of CWB in south-western parts under projected climatic conditions (Figure 3b and 4b).

### Changes in the risk of CWB

The models predict that there will be increases in area suitable for CWB infestation in Chipinge, Chimanimani and Mutare districts by 2080 (Table 4). The greatest increase was found in the Chimanimani district where the GLM projections indicated that the area suitable for CWB will increase by over 200% from 2 697 ha to 8 098 ha. Alternatively, the BRT showed only a 44.7% increase in area for Chimanimani district but projected more than 150% increase in area favoured by CWB in Mutare district. For Chipinge district, the GLM and BRT projected an increase of 30.4% and 12.2% in area favourable for CWB in 2080 respectively. Both models agreed that the suitable area will decrease by 2080 for Mutasa district (Table 4). The GLM showed that the total area with high risk of CWB will decrease in 2080 by 3.9% while the BRT showed that the total area will increase by 14.4%.

## Discussion

### Biotic factors affecting distribution of CWB in Zimbabwe

Precipitation-related factors have been found to be most important in determining the distribution of the CWB with precipitation of the wettest quarter (Bio16) and precipitation seasonality (Bio15) being the most important. The wettest quarter is often January to March in Zimbabwe's tropical savannah climate. These findings agree with Kutywayo [30] who observed that the emergence patterns of adult CWB in Zimbabwe correspond with rainfall, peaking in January. This suggests that areas that have more rainfall are likely to have more CWB as rainfall induces the pest's emergence and beginning of breeding. Precipitation seasonality, which is determined by patterns in the distribution of precipitation such as beginning or end of the season, was also found to be important especially for the GLM.

Since the CWB lifecycle can last up to 24 months [24], the biotic factors that are related to its distribution have to be conducive for CWB for at least two seasons. In light of this, the seasonality of rainfall becomes important as it determines the variation of the supportive environment of the pest over a longer time period. The relationship between weather variables such as temperature and precipitation with the occurrence of CWB could be clearly determined in long-term time series data, which are difficult to obtain or maintain making model based estimation more practical.

Temperature-related factors such as maximum temperature of warmest month (Bio5) and temperature seasonality (Bio4) were found to not be important in determining CWB distribution. This is likely due to the fact that variation in temperature across the coffee zones is relatively small, as temperature (especially range) is one of the most important factors in determining areas that are suitable for coffee production in Zimbabwe [24], [58]. Interestingly, previous studies have found that temperature variability is the most important factor for the distribution of other important pests of coffee such as the coffee berry borer [8], [18]. The observation that precipitation factors are more important than temperature-related factors could indicate that the current and projected temperature ranges are both within range required for CWB survival and propagation. More work is therefore required to determine the thresholds and response of CWB to temperature ranges through behavioural bioassays.

### Projected distribution of CWB and implications on coffee production

Given the projected future climatic conditions, the BRT and GLM models predicted that the risk of CWB will increase in many districts of Zimbabwe; a finding that is consistent with other studies about the impact of climate change on pests in coffee and other crops [2], [7], [11], [12], [15], [16], [59]. Studies on the impact of climate change on the coffee berry borer (*Hypothenemus hampei*) for example, showed that the impact of the pest is likely to increase due to projected increase in temperature from climate change [8], [18]. Some studies that looked at the general suitability of coffee production based on biotic factors projected that the areas suitable for coffee in many countries will decrease due to climate change [12], [14], [16], [60]. These studies did not specify exactly what biotic or abiotic factors will make coffee unsuitable in the studied areas and probably, increased pest pressure could be among the most limiting factor to coffee under projected future climatic conditions production. Our findings concur with general observations that the CWB is increasingly becoming the most important pest of coffee in Zimbabwe and other African countries [28].

It is surprising that both the BRT and the GLM projected that the CWB risk for Mutasa district will decline with some areas that currently have high risks projected to have reduced risk by 2080. Mutasa district had the highest incidence (70.2%) and severity (20.9%) of CWB in biological surveys [28] and is the hub of smallholder farmers in the country. Given that the coffee area in Mutasa is only in Honde Valley, it could be possible that the conditions in the area that are currently supporting the high incidences will become less favourable for the CWB. Although impacts of climate change on agriculture will largely be negative, some areas will become more productive in the wake of climate change due to increased carbon dioxide levels which in turn increases the photosynthetic rates of plants and ability of the plant to produce enough for pests without affecting yields [61]. For example, while some places will have less and unevenly distributed rainfall, some areas will have more rainfall and more conducive environments [59] and this could be the case for Mutasa district. Conversely, climate change could have exceeded the precipitation and temperature thresholds required for survival and spread of the pest leading to the area not being favourable for the pest to thrive and hence reduced risk.

Although the BRT and GLM agreed about the areas and spatial distribution of CWB under both the current and projected climatic conditions, the fact that they differed and in some respects widely show that the assumptions of these models should be examined and tested so that we can confidently develop effective management and mitigation programs. There are fundamental differences in the assumptions and fitting of data between the BRT and the GLM which could partly explain some of the differences in obtained distribution of CWB. The GLM is in the group of traditional straightforward regression based approaches that have been modified for analysis of data with non-normally distributed errors such as presence-absence data [49]. The GLM allows non-linearity in the model structure and thus accommodates the exponential family of distributions such as Poisson, Gaussian, binomial and gamma using link functions. It assumes linear effects on the transformation of the response variable with predictor variables being either discrete or continuous [52], [62], [63]. The BRT model is significantly different from the GLM in approach. Unlike the GLM which is based on statistical methods, the BRT borrows its approach from statistics, data mining and machine learning [40], [49]. Therefore, by design, the BRT is superior in approach than the GLM because the final BRT model is learned from the data and not predetermined as in the GLM. This may therefore explain the partial differences in CWB suitable area and spatial distribution from the two models and the superiority of BRT in performance in this study.

The disagreement of the models in predicting the current distribution of CWB was greatest for the Mutare district. This discrepancy could be the result of the topology of the area. That is, the coffee production zone for Mutare district is on mountainous areas and valleys (Vumba and Burma Valley) where the local rather than the general climate is most significant, sometimes over very small areas. In addition, the district is dominated by large scale corporate farmers who, despite climatic factors being conducive for CWB, have resources for its control and for creating an environment discouraging it through irrigation and other management practices. This is because the CWB was previously regarded as a pest of mismanaged or stressed coffee and thus can distort the relationship between CWB and biotic factors used in the predictions and thus the accuracy of the models.

### Climate change adaptation mechanisms for the CWB management

Given the general increased risk from CWB in the face of climate change, there is a need to develop adaptation strategies in order to minimize the impact of the pest on coffee production and on those who rely on coffee production. Research should focus more on understanding the host preferences of the pest in terms of varieties and agronomic conditions. It has been suggested that coffee agroforestry systems may be an option to counter the impacts of climate change including reducing the risk of pests [3], [8], [17], [64]. Further studies are required to verify the impact of coffee agroforestry systems in reducing the risk of CWB. Crop improvement programmes for coffee could focus on developing varieties that are less preferable to the pest or whose yield and survival are not significantly reduced by pest attack. Our assumption that current coffee production areas will not change may need to be re-examined in the context of these results given that the pressure of serious pests such as CWB may determine sustainability of coffee production, especially for smallholders who owned the majority of the farms sampled.

Other cost-effective and environmentally safe management options should also be looked at, with a focus on areas whose risk will increase due to climate change. For example, research on the potential use of pheromones for monitoring and trapping CWB, the use of biological control agents and alternative insecticides should be strengthened in the face of climate change [26], [65], [66]. Site specific plans and response mechanisms could also be developed given the current and projected changes in the areas and spatial distribution of the CWB in Zimbabwe. In addition, although suggesting growing coffee in low risk areas may have impacts on the livelihoods of many farmers and farm workers, coffee production in Zimbabwe could be more concentrated in the areas with less projected risk of CWB. Other climate change adaptation strategies such as crop insurance schemes, promotion of crop-livestock synergies, adoption of conservation farming methods and diversification of land uses, agricultural systems and sources of income are also suggested for the coffee sector in Zimbabwe[13], [67]. Further work of this nature should also be done for other pests of coffee as well as for other crops of strategic importance for national food security and economic development in order to build national preparedness [68].

### Potential limitations

Modeling species distribution in space and time is based on assumptions inherent in the models, some of which cannot be tested [33], [37], [69]. Future climate projections are also based on assumptions that are very difficult to ascertain. The area highly suitable for CWB obtained from this work does not necessarily translate into a measure of field infestation as, in addition to the biotic factors used in this study, multiple other physical and socioeconomic factors determine CWB incidence and infestation [28]. Given these challenges, the validity of models in making predictions is questioned [37], [69] but modeling still remains an important tool for future planning purposes [36], [37], [69]. The results obtained from these models could be very useful in responding to climate change challenges especially in the agricultural sector which is projected to be the most vulnerable.

## Conclusions

The BRT and GLM models developed in this study were considered reliable in explaining the distribution of CWB. When these models were modified to incorporate how the climate of the region may change by 2080, they predict that CWB will become more common in some coffee districts while in one district this pest may decline in importance. Precipitation related variables were found as the most important predictors of CWB distribution. While discrepancies exist between the degree to which these models predict whether CWB will increase or decrease in different districts, they provide a starting point for planning mitigation programs and strategies to respond to the impacts of future climate change. While not addressed in this study, the future distribution and profitability of coffee production should also be considered when developing strategies for future coffee production. There is need to develop and apply adaptation strategies to minimize the negative impacts not only on the CWB but possibly for other pests.
